# Regenerative therapy by endometrial mesenchymal stem cells in thin endometrium with repeated implantation failure. A novel strategy

**DOI:** 10.5935/1518-0557.20190061

**Published:** 2020

**Authors:** Alberto E Tersoglio, Sebastian Tersoglio, Dante R. Salatino, Matías Castro, Adriana Gonzalez, Mariana Hinojosa, Onias Castellano

**Affiliations:** 1 International Center for Assisted Reproduction, Mendoza, Argentina

**Keywords:** endometrial mesenchymal stem cells, thin endometrium, repeated implantation failure, chronic endometritis, endometrial flow cytometry, endometrial histopathology

## Abstract

**Objective:**

Our primary objective was to evaluate the endometrial changes before and after the transfer of endometrial mesenchymal stem cells (enMSCs) in a population of thinned endometrium women, with absence or hypo-responsiveness to estrogen and repeated implantation failure (RIF). The secondary objective was to evaluate the clinical outcomes of the intervention in terms of clinical pregnancy (CP), early abortions, ongoing pregnancy and live birth delivery rate (LBDR) per *in vitro* fertilization (IVF) cycle.

**Methods:**

A longitudinal and experimental study. The intervention was defined as "subendometrial inoculation of enMSCs," and the post-intervention changes were evaluated by the following variables: endometrial thickness (Eth), endometrial flow cytometry (enFC), endometrial histopathology (enHP) and endometrial immunohistochemistry (enIHQ). The variables were analyzed after the intervention (Post-treatment) regarding previous values (Pretreatment).

**Results:**

Eth values before and after treatment with enMSCs were 5.24±1.24 mm vs. 9.93±0.77 (*p*=0.000), respectively. Endometrial Flow Cytometry showed significant differences in favor of Normalized variables in the post-treatment assessment, associated with the pretreatment, LT/Li, LB/Li, NK/Li, CD8/CD3^+^ and CD4/CD8 (*p*≤0.015), respectively. Only two variables Li/PC and CD4/CD3 had NS (*p*=0.167 and 0.118). A similar analysis was performed on enHP with an HP increase post-treatment (*p*=0.007). The CP rate was 79.31% (23/29), a live birth delivery rate per embryo transfer was 45.45% (10/22) and ongoing pregnancy 7/29 (24.14%).

**Conclusion:**

Subendometrial enMSCs inoculation produces a significant increase in endometrial thickness; normalize the enHP, enIHQ and enFC. As a result, IVF after treatment with enMSCs yields a higher rate of CP and LBDR.

## INTRODUCTION

One in nine couples in Europe and the USA is affected by implantation disorders, and it is estimated that RIF has a prevalence of 15-20% in IVF (Teklenburg *et al*., 2010; Cicinelli *et al*., 2008). There is enough evidence that appropriate Eth is essential for a successful pregnancy (Kovacs *et al*., 2003), and a thin endometrium is associated with low pregnancy rates (El-Toukhy *et al*., 2008; Glissant *et al*., 1985; Dickey *et al*., 1992). The optimal endometrial thickness (Eth) for conception remains controversial among clinicians. Eth less than 7 mm on ultrasound is generally considered sub-optimal for embryo transfer (Eftekhar *et al*., 2018). Approximately 0.6-0.8% of patients do not reach minimum thickness for embryo transfer (Al-Ghamdi *et al*., 2008). It has been stated that the probable causes of thin endometrium are as follows: inflammatory causes (acute or chronic endometritis/CE); iatrogenic (repeated curettage, polypectomy; hysteroscopic (myomectomy or laparoscopic) where the cavity is opened, and the irrational use of clomiphene citrate (Mahajan & Sharma, 2016). It is also indicated that thin endometrium may be a result of an individual uterine structural pattern (Scioscia *et al*., 2009).

In spite of the large variety of treatment, most of the choices achieve only minor modifications in endometrium thickness and have not been validated (Eftekhar *et al*., 2018). Angiogenesis is the formation of new blood vessels from existing vascular structures by elongation, intussusception or sprouting of endothelial cells; and after birth, vascularization is determined and maintained by angiogenesis (Ahmed ( El-Badri, 2017). Physiological angiogenesis does not occur in most organs in the adult. However, endometrium is the site where normal angiogenesis take place, and it is a fundamental process in the menstrual cycle as well as in embryo implantation (Gordon *et al*., 1995). Rogers *et al*. (1998) suggested that endometrial angiogenesis occurs by elongation and intussusception rather than sprout formation. Blood vessels consist of an inner endothelial cell layer lining the vessel wall and perivascular pericytes, also known as mural cells, which envelop the vascular tube surface.

Pericytes are multipotent cells that are heterogeneous in their origin, function, morphology and surface markers (Ahmed ( El-Badri, 2017). Analysis of the anatomic relationship between pericytes and endothelial cells shows that they interact closely via juxtacrine or paracrine signaling (Gaengel *et al*., 2009). Pericytes were reported to respond to platelet derived growth factor receptor-Β(PDGF-Β) and transforming growth factorΒ(TGF-Β) which are released by platelets after an injury (Mills *et al*., 2013). This chemotactic response to PDGF-Βleads to the migration of pericytes to the outer layer of the blood vessel. This migration enables the endothelial cells to proliferate at the wound site in response to vascular endothelial growth factor-VEGF (Möhle *et al*., 1997). Angiogenesis plays a key role in endometrium remodeling, being the vascular endothelial growth factor (VEGF) an important regulator of this process. A number of studies have reported that VEGF is expressed differentially in thin endometrial (Sugino *et al*., 2002; Kashida *et al*., 2001; Sharkey *et al*., 2000; Miwa *et al*., 2009).

Uterine NK cells (uNK) are a major source of cytokines and angiogenic growth factors (Lash *et al*., 2006) including VEGF-A, placental growth factor (PLGF), and angiopoietin (Trundley & Moffett, 2004), which may produce cytokines to promote angiogenesis during embryo implantation. Although uNK cell counts are increased in women with recurrent miscarriages (RM) and RIF, angiogenesis seems to be paradoxical in the two groups of women, namely increased in RM and reduced in RIF (Chen *et al*., 2017a). Previous studies have shown that isolated CD56+ uNK cells from women with RIF produce lowest levels of angiogenic factors, such as VEGF, PLGF, PDGF-BB, compared with women with RM and fertile controls (Chen *et al*., 2016). It has been proposed that angiogenesis may be reduced by stimulating the STAT5 pathway in uNK cells in women with RIF (Chen *et al*., 2017b). There is sufficient evidence of the existence of SCs in the human endometrium and the likelihood that they are a therapeutic resource in endometrial atrophy, thinned endometrium and Asherman syndrome (Singh *et al*., 2014). The skill to maintain a normal karyotype after several passages (Allickson *et al*., 2011), the ability to differentiate into multiple cell lines under standard culture (Rossignoli *et al*., 2013; Gargett *et al*., 2016), its immunosuppressive properties (inhibits LT, LB and NK) (Ribeiro *et al*., 2013), make endometrial mesenchymal cells (enMSCs) a source of excellence in certain regenerative therapies. These immunomodulatory properties are explained by the release of inflammatory cytokines in the tissue (Bernardo & Fibbe, 2013).

On the other hand, the low immunogenic capacity and tumor genicity makes it the choice in clinical application (Zhou *et al*., 2011). An important concept is that both infection and inflammation may inhibit regeneration of traumatized endometrium through damage to the stem/progenitor cells by effector molecules, which also contribute to the deposition of fibrotic tissue (Gargett & Ye, 2012). Oocyte donation cycles are ideal to measure the independent effect of Eth as a parameter of endometrial receptivity, because there is lower variability in embryo quality. RIF is a clinical entity which refers to a situation when implantation has repeatedly failed to reach a stage recognizable by pelvic sonography. It represents a very frustrating condition for both the healthcare professional and the patient.

In order to isolate the endometrium as the main responsible for RIF, different embryonic condition and a number of consecutive failed IVF cycles have been proposed. There is a tacit acceptance of defining RIF as the impossibility of obtaining clinical pregnancy after three consecutive IVF attempts, in which one to two embryos of high-grade quality are transferred in each cycle (Simon & Laufer, 2012). The precise definition remains controversial, so other suggestions have been proposed (Polanski *et al*., 2014). Today, the most accepted definition of RIF, is a failure to achieve a clinical pregnancy after the transfer of three or more good-quality embryos in women <35years of age, and four or more good quality embryos in women (35years during fresh or frozen embryo transfer cycles (Coughlan *et al*., 2014). In the presence of RIF of endometrial origin, the chronic endometritis (CE) and limited immunological alterations are plausible for specific treatments (Coughlan *et al*., 2014). In women with chronic endometritis (CE), the endometrial immune responses are often shifted towards pro-inflammatory profiles and are consequently unfavorable to invading embryos (Park *et al*., 2016). The high association between Chronic Endometritis (CE) and RIF is established (14%-31%), of unknown etiology (28%) and recurrent pregnancy loss (9-13%) (Johnston-MacAnanny *et al*., 2010; Cicinelli *et al*., 2005; Tersoglio *et al*., 2015; Bouet *et al*., 2016; Kotaro *et al*., 2017). A recent publication shows that 34.4% of women with RIF have CE, which is higher than those of women with recurrent pregnancy loss or fetal death (Wang *et al*., 2019).

The impact of chronic endometritis on perinatal outcomes has been considered, taking CP and LBR with/without treatment (56*vs*.7%) into account (McQueen *et al*., 2014). It has been proven that CE yielded significantly decreased TGF-Β and IL-10 expression in the endometrium, which reflects T-regulatory (Treg) cells in number or functional deficiency (Wang *et al*., 2019). EC resolution is of such complexity that the bacteriological clearance is inefficient as a criterion of cure, which could explain the high rate of persistent CE found (24.6 and 17.6%) (Cicinelli *et al*., 2015; Cicinelli *et al*., 2018).

Our primary objective was to evaluate the endometrial changes before and after enMSCs transfer in a thinned endometrium population, with absence or hypo-responsiveness to estrogen. The secondary objective was to evaluate the results of the intervention in terms of CP, early abortions, ongoing pregnancy and live births in IVF cycles.

## MATERIAL AND METHODS

It is a longitudinal and experimental study focused on the endometrium (Salatino, 2019). The intervention was defined as "subendometrial inoculation of enMSCs" and the post-intervention changes were evaluated by the following variables: Eth (mm), enFC and enHP. The variables were analyzed after the intervention (Post-treatment) in reference to the previous values (Pretreatment). A Binomial distribution was obtained through the dichotomization of the enHP and enCF (Normal or Abnormal) variables, the latter in relation to the 95% CI values of reference.

### Definition of thinned endometrium and RIF

Thinned endometrium was considered to be the one that after twenty days of estrogen supplementation with an 8 mg/day dose of 17Βestradiol did not reach at least 7 mm of Eth, measured in the midsagittal plane by transvaginal ultrasound (TVU). In ovulatory patients the endometrial thickness was checked on the day of LH and confirmed under the regimen described beforehand. We consider RIF as the absence of implantation after three or more cycles of IVF/ICSI or cryotransfer, where the cumulative number of embryos transferred was no less than three blastocysts with high quality 311-411 or 511 of Gardner-Schoolcraft, the latter under the Istanbul criteria (Alpha Scientists in Reproductive and ESHRE special interest group of Embryology, 2011).

### Patient selection

We selected 29 patients with thin endometrium, hypo-responsive/unresponsive to estrogens, with RIF. The inclusion criteria were absence of uterine malformation; autoimmune thyroid disease; thrombophilia; polyps; hydrosalpinx; and those who did not accomplish enHP, enIHQ and enFC pre/post-treatment; with the presence of normal uterine cavity ascertained by hysterosonography or hysteroscopy. In 23/29 (79.31%)there were oocyte donations and6/29 (20.69%) autologous cycles. The age of patients (in years), number of previous IVF cycles, body mass index and length of sterility (year) were calculated with Mean±DS and (interval); 41.72±5.18 (32-53),4.03±1.65 (3-8), 24.84±5.09 (19-40), 13.66±5.81 (5-20), respectively. The measure of basal Eth (mm) was 5.24±1.24 (2-6.9). In 6/29 (20.69%) they presented a history of uterine interventions (myomectomy=3, septum resection=1 and repetitive curettage=2). 6/29(20.69%) patients showed CE after being with specific antibiotics, in whom IMH was normalized. The basal pretreatment histopathology was normal in 6/29 (20.69%), and 23/29 (79.31%) presented abnormal patterns (Table 1).

**Table 1:** Demographic, patient and basal clinical characteristics

Number of cycles/patient	29
Number of previous IVF cycles	4.03±1.65 (3-8)
Body Mass Index (Kg/m^2^)	24.84±5.09 (19-40)
Patient age (year)	41.72±5.18 (32-53)
Length of sterility (year)	13.66±5.81(5-20)
Basal endometrial thickness (mm)	5.24±1.24 (2–6.90)
Nº Patient w/ Previous Uterine Interventions	6/29 (20.69%)
Nº Patient w/ Previous CE by IHQ	8/29 (27.59%)
Oocyte donation cycles	23/29 (79.31%)
Homologous cycles	6/29 (20.69%)
Endometrial biopsy	
Normal	6/29 (20.69%)
Abnormal	23/29 (79.31%)

Note: Values were represented by mean ± DS. (Interval or %) where correspond

### Endometrium Thickness and Endometrial Biopsy

The endometrial thickness was measured in the middle sagittal section by transvaginal sonography (TVS), using a volumetric vaginal probe. The endometrial thickness in pre/pos-treatment was the highest value achieved under estrogen therapy upon progesterone administration onset. We obtained at least two endometrial biopsies from each patient, the first prior to treatment and the second after treatment with enMSCs. All biopsies were carried out under hormone replacement therapy (HRT), starting with 17( estradiol 8mg/day per os, for at least 20 days. The progesterone gel is added in an intravaginal dose of 180 mg/day. The biopsies were made using the Cornier's pipelle, with axial movements on the entire endometrial surface in order to ensure an ideal harvesting.

### Histopathological and bacteriological criteria for normal and abnormal endometrium

**Figure 1:**
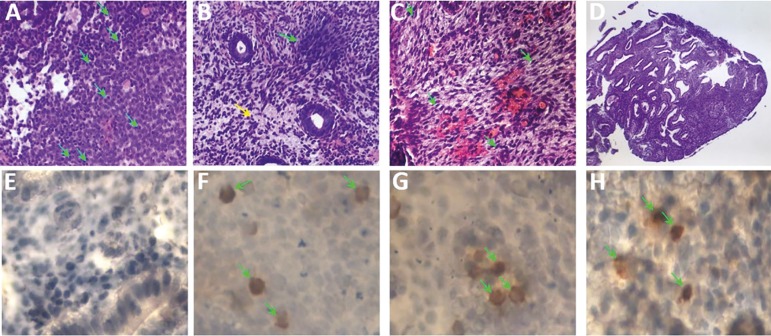
Staining of tissue samples showing (**A**) Plasmatic Cells, (**B**) Stromal edema and elevated Stromal density, (**C**) Spindle Stromal cells, (**D**) micropolyps, (**E**) Normal CD138, (**F**) High CD138, (**G**) High CD56 and (**H**) High CD20

The histopathological criteria for dating were those corresponding to Noyes *et al*. (1950). In case of a CE history, the pretreatment biopsy must accomplish the bacteriological clearance with an endometrial stromal plasmocyte density index (ESPDI(0.25) as a cure criterion (Akopians *et al*., 2015; Kitaya *et al*., 2017). ESPDI was the result from the sum of the stromal CD138 cell counts divided by the number of HPFs evaluated. The following histopathological criteria were considered for an abnormal endometrium: 1) lymphocyte clusters, 2) polymorphic inflammatory cells (stromal, superficial epithelium and intraglandular), 3) superficial mucosal edematous change, 4) presence of fibroblast-like stromal cells (spindle stroma) (Check *et al*., 1991), 5) high-density stromal cells, 6) pseudo-stratification and mitotic nuclei in both glandular and surface epithelial cells, 7) micro polyps, 8) delayed endometrium differentiation (out of phase). Having no more than three of the previously established criteria was required as a post-treatment standardization, as per depicted in Figure 1.

The bacteriological test consisted of a freshtest with Gram/Giemsa staining and culture in Thayer Martin medium, sheep blood agar, and chocolate agar, Agar medium Saboreaud, and EMB/CLDE of vaginal and endocervical samples, and endometrium washing with PBS. Chlamydia trachomatis was tested using immunofluorescence; Mycoplasma hominies and Urea plasma urealyticum using Mycofast-urea/arginine. All the cases included in the present study required negative bacteriology before the enMSCs treatment.

### Endometrial Multicolour Flow Cytometry

A four colors and six parameters FACS Calibur two lasers, (Bexton Dickinson(r)) was used for cytometric evaluation. The following variables were considered: Li/PC (total lymphocyte/total cell population ratio), T-lymphocytes (LT), B lymphocytes (LB) and NK (NK) cells over Li; CD3+ CD4+, CD3+ CD8+, CD4/CD8 ratio and NK CD56/CD16 subpopulations. We established a normal reference group for the cytometric variables (n=25), corresponding to oocyte donors with a normal reproductive history (absence of abortions, with normal live births, no history of vaginosis, negative endometrial bacteriology and normal endometrial pathological anatomy) (Table 2). The values obtained in this study were considered for each variable as normal or abnormal, as per related to the reference group.

**Table 2:** Endometrial Flow Cytometry. Normal Reference Value

Variables - *n = 25*	Mean ± 2SD	95% CI	*p* value
LT/Li	51.96±16.05	35.908 - 68.01	0.112*
NK/Li	40.04±14.58	25.458 - 54.62	0.157*
CD4/Li	44.32±7.78	36.539 - 52.1	0.324*
CD8/Li	56±12.31	43.6912 - 68.31	0.729*
CD4/CD8	0.64±0.38	0.2676 - 1.01	0.137*
Li/Cells population	5.6^‡^	2 - 6.27	0.000^†^
LB/Li	2^‡^	1 – 2.98	0.000^†^

* *p*>0.05 Normal distribution;

† *p*<0.05 No Normal distribution (by Shapiro Wilk test);

CI= Confidence interval;

SD = Standard deviation;

‡ Median

### Cell isolation and culture

The endometrial tissue was dissociated using enzymatic and mechanical dissociation, and separated into stromal single-cell suspensions and epithelial clumps as previously described with several modifications (Allickson *et al*., 2011; Akopians *et al*., 2015; Chen *et al*., 2016). Endometrial tissue samples were washed in DMEM/F-12 w/15mM HEPES buffer (Gibco,#11330-032), 5% newborn calf serum (Invitrogen), and 1% antibiotic-antimycotic, (Gibco,#15240-062) (Bench Medium), then weighed and cut into small pieces <1 mm^3^. The tissue fragments were digested with 0.5% (wt/vol) type IV collagenase (Gibco,#17104-019) and 40µg/ml type I deoxyribonuclease (Worthington Biochemical Corporation) in DMEM/F-12 for 1.5h at 37°C in a stirring machine. Typically, 10 ml of the cell dissociation medium was used per 1g tissue. The dissociated cells were filtered through a sterile 40-µm cell strainer (BD Biosciences, Durham, NC, USA).

Most of the stromal cells and blood cells were present as a single-cell suspension, passed through the cell strainer into a sterile 50-ml polycarbonate tube, whereas the undigested fragments, mostly comprised of glandular clumps, were retained in the strainer. Stromal single-cell suspensions were layered over Ficoll-Paque PLUS (GE Healthcare Bio-Sciences AB, #17-1440-02) and centrifuged to remove the red blood cells. The medium/Ficoll interface, mainly containing stromal cells and peripheral blood mononuclear cells, was carefully aspirated, washed with Bench Medium, and then subjected to culture. Endometrial stromal cells were seeded at a clonal density of 50-100 cells/cm^2^in two flasks of 75 cm^2^ and 25 cm^2^ (Biofil#TCF011250,TCF011050) with DMEM/F-12 plus wo/Hepes (GIBCO, 11320-033) supplemented with 10% newborn calf serum (Invitrogen), 5% Anti-Anti (Gibco) and 2mM Glutamine (Invitrogen) up to obtain total confluence. When the cells reached 75% confluence, they were detached using a 0,125% trypsin solution (GIBCO,#27250-018), diluted and plated onto new T flasks. The cells were sub-cultured by the third passage and cryopreserved (Amable *et al*., 2013a).

### enMSC culture immunophenotyping

In the confluent stage, the cells were characterized by the flow cytometry, previously marked using the Human MSC analysis Kit (BD biosciences#562245) (CD19, CD34, CD45, CD73, CD90, CD105, HLA-DR). Under the recommendations of the International Society for Cellular Therapy, MSCs should be positive for CD73, CD90 and CD105, but negative for CD34, CD45, CD11b or CD14, CD19 or CD79(, and HLA-DR. Additionally, SUSD2/W5C5 and CD140b (PDFGF-RΒ^+^) (BD biosciences, # 558821, 566657, respectively)were used as a positive marker. When the cell population reached the degree of confluence, usually in passage 5 to 7 and the cytometry results show less than 0.3% for CD19, CD34, CD45 and HLA-DR, and more than 99.9% for the markers, CD73, CD90 and CD105 were transferred under sonographic control using a transvaginal probe. In all cases, enMSCs transfer was carried out in the estrogenic phase under estrogen supplementation for 6 to 8 weeks.

### enMSCs cryopreservation

The residual flasks were cryopreserved in a suspension solution with 50% DMEM/F12, supplemented with 40% Fetal Bovine Serum (Internegocios, Argentina) and 10% DMSO (MP Bio, USA). The cells suspended in the medium were frozen at -4°C for one hour and then at -70°C for 24 hours and finally transferred to the N2 tank in cryotubes. In the warm-up phase, the tubes are transferred to a water bath at 37°C and resuspended with the DMEM/F12 solution with 10% FBS and centrifuged at 1,200 rpm for 7 minutes. Viability was evaluated with 0.4% Tryptan Blue (SIGMA#T8154) in PBS.

### Platelet-Rich Plasma Preparation

The plasma platelets concentrate obtained by centrifugation of the patient's whole blood was named PRP and this was applied as a diluent for the enMSCs. The peripheral blood was collected using tubes containing 3.2% sodium citrate solution. The preparation protocol was divided into two centrifugation steps. In the first centrifugation, the relative centrifugal force applied was 300×g for 5 min at 18°C. The whole plasma above the buffy coat was collected (PRP1) and transferred to a new tube. The second centrifugation step used 700×g for 17 min at 18°C. The platelet-poor plasma (PPP) was transferred to a new tube. The platelet pellet obtained from 1 ml of PRP1 was resuspended in 300µl of PPP (PRP2). Platelet activation was induced by adding 20 mM CaCl2 and 25 IU/ml human thrombin incubated at 37°C for 1h or at 4°C for 16h. Finally, for recovering the activated PRP2, the samples were centrifuged at 3000×g for 20 min at 18°C and the supernatant (activated PRP2) was collected by aspirating Activated PRP, hereafter referred to as PRP, and frozen at -20°C until use (Amable *et al*., 2013a; 2013b).

### Transmiometrial transference of enMSC

Before transferring, the enMSCs is harvested using a 0.125% trypsin solution (GIBCO, #15090-046), washed with PBS and re-suspended in PBS containing 2% FBS. Viable and total cell numbers were determined using Trypan blue in Neubauer chambers. The enMSCs were transferred in a number of 2.5 to 3.6.10^6^ cells, diluted in 1 ml of autologous PRP with the use of an Embryo Transfer Transmyometrial catheter (COOK # K-TTET-19-32.5) after sedating the patient.

### Embryo laboratory, transfer, receptor protocol

Having ovarian activity, the hypothalamus is suppressed with the use of depot GnRHa, Triptorelin 3.76 mg in a single dose, or Leuprolide acetate in doses of 200 to 300 micrograms/day, in a long regimen; beginning the estrogen replacement in the presence of a plasmatic estradiol <30 pg/ml. Both the Estradiol valerate and the 17 β-estradiol were administered in increasing doses, orally, according to the endometrial response and established protocol. Five or six days before the transfer, we added progesterone gel in daily doses of 90 mg per vaginal administration. In cases of inadequate endometrial response, we associated transdermal patch replacement with variable doses (50-150 micrograms every 2.5 days). All transfers were individualized according to previous biopsies and level of IHQ hormonal receptors. Only expanded or hatched optimum quality blastocysts (score 4.1.1 or 5.1.1) were transferred in a number no higher than two. In cases where a second blastocyst was of suboptimal quality (412, 421, 512 or 521) the transfer was made. In cases of a single embryo, the transfer (SET) was considered only when it were of optimal quality. All of the embryos at day 5 were cryopreserved and transferred at the time of endometrium optimization (Frozen-thawed embryo-transfer).

### Statistical Analysis

Statistical analyses were performed using the SPSS version 20 (IBM) and the STATA statistical software version 14 (StataCorp). The data distribution was checked using the Shapiro-Wilk test. For the normal reference group, the 95% CI was calculated with the mean(2DS for the normal distribution data; median and range for skewed data. The endometrial thickness was compared in the pre and post-treatment using the paired samples t-test; for the enFC and enHP the McNemar test was applied. The level of significance was set at *p*<0.05.For the enFC, each variable was dichotomized as normal or abnormal vis-à-vis the reference values. Histopathology was normal in the post-treatment when a (3 abnormal pattern was observed.

## RESULTS

**Figure 2:**
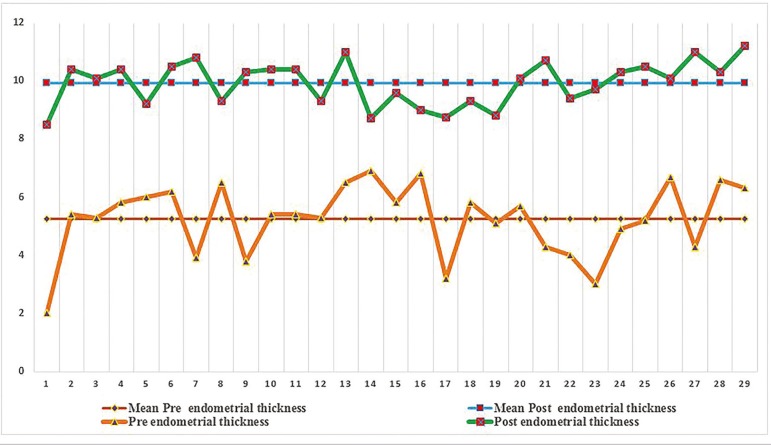
Individual evolution of endometrial thickness

A total of 29 cycles/patients were analyzed with a mean age of 41.03±4.52, with a history of infertility for 13.66±5.81 years, and 3.79±0.73 previously failed IVF cycles; all with thin endometria. As depicted on table 3, the Eth value between pre and post-treatment with enMSCs resulted in 5.24±1.24 mm *vs*. 9.93±0.77 (*p*=0.000), respectively. As displayed in Figure 2, the endometrial thickness evolution on a case-by-case basis is represented individually in relation to the intervention with enMSCs, ratifying an increase of more than 8 mm in all cases.

As you can see in Table 3, after the dichotomization of the enFC variables (Normal or Abnormal), the lymphoid populations studied by FC showed significant differences in favor of Normalized variables in post-treatment. As a result: LT/Li, LB/Li, NK/Li, CD8/CD3^+^ and CD4/CD8 ratios were normalized, *p*=0.013, 0.002, 0.049, 0.000, 0.000, respectively. Both variables (Li/PC and CD4/CD3^+)^ show a post-intervention increase, but it turns out to be NS (*p*=0.167 and 0.118). Analyses of the enHP variables were carried out according to the criteria previously described. We found a normal increase in post-treatment (*p*=0.007).

**Table 3:** Evolution of Endometrial Variables Pre and Post Treatment eMSCs

Variables	PreSC_s_	Post SC_s_	*p* value
Endometrial Thickness	5.25±1.24	9.93 ± 0.77	0.000*
Endometrial Flow Cytometry			
Li/PC			
Normal	13/29 (44.8%)	20/29 (69%)	0.167^†^
Abnormal	16/29 (55.2%)	9/29 (31%)	
LT			
Normal	15/29 (51.7%)	26/29 (89.7%)	0.013^†^
Abnormal	14/29 (48.3%)	3/29 (10.3%)	
LB			
Normal	8/29 (27.6%)	18/29 (62.1%)	0.002^†^
Abnormal	21/29 (72.4%)	11/29 (37.9%)	
NK			
Normal	14/29 (48.3%)	23/29 (79.3%)	0.049^†^
Abnormal	15/29 (51.7%)	6/29 (20.7%)	
CD4			
Normal	14/29 (48.3%)	21/29 (72.4%)	0.118^†^
Abnormal	15/29 (51.7%)	8/29 (27.6%)	
CD8			
Normal	13/29 (44.8%)	25/29 (86.2%)	0.000^†^
Abnormal	16/29 (55.2%)	4/29 (13.8%)	
CD4/CD8			
Normal	13/29 (44.8%)	28/29 (96.6%)	0.000^†^
Abnormal	16/29 (55.2%)	1/29 (3.4%)	
Endometrial Histopathology			
Normal	6/29 (20.7%)	17/29 (58.6%)	0.007^†^
Abnormal	23/29 (79.3%)	12/29 (41.4%)	

* T-test paired samples,

† McNemar Test.

As it is shown in Table 4, over 29 females with embryo transfer, 23/29 (79%) were clinical pregnancies, from which there were live births 10/22 (45.5%) and 7/29 (24.14%), respectively, continued with their pregnancies with gestational ages greater than 17 weeks. The implantation rate resulted in 68.12%, with a mean value of 1.86±0.34 embryos transferred per patient. With a prematurity rate of 1/10 (10%) and single delivery 7/10 (70%).

**Table 4:** Clinical and Pregnancy Outcome

n	29
Nº of Embryo Transferred (mean±SD)	1.86±0.34
Implantation Rate (%)	68.12%
Clinical Pregnancy (%)	23/29 (79.31%)
Abortion Rate (%)	6/29 (20.69%)
Ongoing Pregnancy (%)	7/29 (24.14%)
Live Birth Delivery Rate (%)*	10/22 (45.45%)
Single (%)	7/10 (70%)
Double (%)	3/10 (30%)
Premature Delivery	1/10 (10%)
Birth weight< 2500 gr	5
Birth weight> 2500 gr	8

* For Initiated Cycles

## DISCUSSION

Thin endometrium may be conditioned by multiple factors, its management should be cause-related, with the aim of increasing endometrial receptivity and simplifying implantation (Lebovitz & Orvieto, 2014). EnMSCs, for their properties of high clonality, multipotentiality, regenerative capacity, immunomodulatory, angiogenic and low immunogenicity are proposed as an alternative in severe endometrial lesions. Adequate uterine vascularity and the regulating cells/factors are needed at the time of implantation, while inappropriate endometrial angiogenesis and immunity may lead to reproductive failure, especially in recurrent miscarriage and RIF (Chen *et al*., 2017 a). A number of non-cellular treatments have been tried to increase endometrial development, but none has been validated so far. Endometrial stem cell research is gaining momentum and the knowledge generated may be translated into the clinical setting within the next decade (Gargett & Ye, 2012). One of the points to be clarified is to define the cell line (s) that will be applied to the study, taking into account the limitations inherent to the method. The enSCs are a heterologous population that includes: mesenchymal stem cells (MSCs), epithelial stem cells (ESCs), endometrial side population (ESP) and endometrial regenerative cell (ERC) (Azizi *et al*., 2018).

The Endometrial Side Population (ESP) are a mixed population, comprising predominantly of precursors of endothelial cells (Masuda *et al*., 2010), and epithelial and stromal cells (Cervelló *et al*., 2010). The proportion of SP cells in whole and fractions of epithelial and stromal cells fluctuate from 0.06-6.2% and 0.01-3.8%, respectively. The SP cells have the capability to extrude the DNA binding dye Hoechst 33342 via the ATP-binding cassette (Cervelló *et al*., 2010). They also exhibit high clonogenicity (Tsuji *et al*., 2008), telomerase activity (Cervelló *et al*., 2010), but the most important and controversial issue is the heterogeneity of SP cells (Masuda *et al*., 2015). Specific markers of human endometrial MSC have been identified. Coexpression of CD140b(PDFGF-RΒ^+^) platelet-derived growth factor-receptor beta (Schwab & Gargett, 2007), and W5C5 have been used to isolate endometrial mesenchymal stem cells (Masuda *et al*., 2012). The gene profile of this CD 146^+^ PDFGF-RΒ^+^ population indicated that these cells expressed pericyte markers, and genes associated with angiogenesis/vasculogenesis, steroid hormone/hypoxia responses, inflammation, immunomodulation, and signaling pathways associated with MSCself-renewal and multipotency (Gargett & Ye, 2012).

Epithelial progenitor cells (ESCs) is a small population with high proliferative potential, that differentiated into large gland-like structures (Nguyen *et al*., 2017). N-cadherin as a marker of epithelial progenitor cells can play a role in endometrial proliferative disorders like adenomyosis, endometriosis, and thin dysfunctional endometrium (Mills *et al*., 2013). The main limitation of EPCs recovery is their location, because they are placed near the basal gland fundus and close to the myometrium. Because of that, it is seldom observed in cell culture. Endometrial Regenerative Cells (ERC) have some features similar to MSC including the capability to modulate the immune system and stimulate Treg production. In this series, phenotyping indicates a predominance of MSCs, but probably due to sharing markers, the population has ESCs and ESP components. Actually, clinical application may not necessarily need a pure population of Stem Cells. However, for stem cell biology and understanding endometrial physiology, it is necessary to obtain single line cells (Masuda *et al*., 2015). A normal reference group was established for the cytometric variables (n=25) in the absence of previously published data. All the variables were analyzed in the normality of their distribution and the 95% CI was calculated in order to increase data precision.

There was a highly significant increase in endometrial thickness after the inoculation of enMSCs, expressing the high regenerative capacity of the intervention. In 8/29 (27.5%) cases, they presented pretreatment values with critical values of <4 mm, despite having been subjected to estrogen therapy for over 20 days. It is interesting to note that in most of the previous IVF treatments the endometrium had not been isolated as the main cause of the repeated failures, even though the majority of the medical therapies had been tried (high doses of estradiol, HCG, aspirin, sildenafil, vitamin E and granulocyte colony stimulating factor). Endometrial lymphoid population normalization should be a primary objective in any treatment where the implantation conditions have been modified. Endometrial NK cells are the major leucocytes source present in the endometrium. It has been established that 47% of RIF presented uNK cell counts outside the reference range, with a majority above the range and a smaller proportion below the range (Chen *et al*., 2017b). In the present study, baseline FC showed in all cases a profound alteration of the lymphoid population and a significant normalization after enMSC. In this series a higher percentage of cytometric variables have been normalized, demonstrating the powerful modulator effect of enSCs. It is also interesting to see that in the subpopulation with CE background a high proportion shows alterations in the leukocyte population and the enMSCs were able to normalize 4/8 (50%); the totality of normalized cases resulted in live births. In contrast with the rest of the CE, none normalized after treatment with enMSCs, where 3/4 cases failed pregnancy. The special behavior of uNK and its implication in angiogenesis have been indicated in previous studies, those who have shown that isolated CD56^+^uNK cells from women with RIF produce lower levels of angiogenic factors, such as vascular endothelial growth factor (VEGF), placental growth factor (PLGF), platelet-derived growth factor (PDGF-BB), compared with women with RM and fertile controls (Chen *et al*., 2016). Additional statistical treatment showed that the normalization of enNK increased significantly the CP and LBDR when compared with no normalization (65.5% *vs.* 13.8% and (44.8 *vs.* 13.8%), respectively.

The CD4/CD8normalization ratio accomplished the highest CP and ongoing/LBDR (79.3% and 58.6%). A high abortion rate (20.69%) was found, which indicates that there are other factors to be considered and cannot be explained by de variables analyzed. The strength of this study lies in the selection of the endometrium as an object of study and the measure of its effects in clinical results. The weakness of this study is that it failed to achieve a complete immunophenotyping of enMSCs and not angiogenic markers. Finally, the endometrium thickness and the standardization of histopathology and immunohistochemistry in post-treatment with enMSCs resulted in higher clinical pregnancy rates in a population with repeated implantation failures, representing a reliable strategy in Assisted Reproduction.
